# Edward O. Bixler, PhD: from the Apollo project and chimpanzees to sleep epidemiology

**DOI:** 10.1093/sleepadvances/zpae020

**Published:** 2024-04-01

**Authors:** Julio Fernandez-Mendoza, Susan L Calhoun, Edward O Bixler

**Affiliations:** Sleep Research & Treatment Center, Department of Psychiatry and Behavioral Health, Penn State College of Medicine, Hershey, PA, USA; Sleep Research & Treatment Center, Department of Psychiatry and Behavioral Health, Penn State College of Medicine, Hershey, PA, USA; Sleep Research & Treatment Center, Department of Psychiatry and Behavioral Health, Penn State College of Medicine, Hershey, PA, USA

**Keywords:** cardiovascular risk, cohort studies, epidemiology, insomnia, pharmacology, psychiatry, sedative-hypnotics, sleep apnea

## Abstract

What an honor to write about Dr. Edward O. Bixler’s contributions to the sleep field. In 1967, Dr. Bixler published a case report on a chimpanzee with implanted brain electrodes while working at an Air Force base in New Mexico. A few years later, in 1971, he published on the sleep effects of flurazepam in individuals with insomnia together with Dr. Anthony Kales, data that he had collected when the Sleep Research & Treatment Center (SRTC) was housed at the University of California Los Angeles. Dr. Bixler, a meticulous scientist, learned from Dr. Kales, a devoted clinician, to study “the whole patient, and all aspects of sleep,” a legacy that continued when the SRTC moved to Penn State in Hershey. Indeed, Dr. Bixler’s tenure at Penn State from 1971 until 2019 kept the science of the SRTC focused on that premise and helped translate scientific evidence into clinical care. He not only contributed early to the pharmacology of sleep and the effects of hypnotics, but he was also a pioneer in “sleep epidemiology.” His “Prevalence of sleep disorders in the Los Angeles metropolitan area” study of 1979 was the first rigorous epidemiological study on sleep disturbances. Starting in 1990, he established the Penn State Adult Cohort to estimate the prevalence and natural history of sleep-disordered breathing and other sleep disorders in adults. Inspired by life-course epidemiology, he established in 2001 the Penn State Child Cohort to estimate the same phenomena in children. This Living Legend paper captures and highlights Dr. Bixler’s enduring legacy to sleep science.

**Statement of Significance**

This is a narrative review of Dr. Edward O. Bixler’s research career and significant contributions to sleep science. His tenure spans from the late 1960s until the year before the COVID-19 pandemic hit. From monitoring and scoring sleep in a chimpanzee to understand the effect on sleep of the rapid decompression experienced by astronauts, to spending 20 years of his career studying the effects of hypnotics and comprehensively characterizing sleep disorders, and to devoting another 30 years of his career to the epidemiology of sleep and its disorders across the human life span.

Our goal in writing this Living Legend tribute is to honor Dr. Bixler, a sleep researcher who has dedicated over 40 years to the field, and highlight his significant and enduring contributions to sleep science. Here, you will learn about Dr. Bixler’s career development, how he got to meet Neil Armstrong and, years later, Dr. Anthony Kales, how he made significant contributions to the systematic study of hypnotic medications and was one of the founding fathers of the field of sleep epidemiology. In addition, you will learn how Dr. Bixler had envisioned “passing the baton” to the next generation of SRTC researchers; ensuring the continuation of his epidemiological legacy.

## Something Called Industrial Psychology (1959–1962)

Dr. Bixler graduated from Occidental College with a major in Psychology and Introductory Statistics in 1959. He furthered his education by obtaining a Master of Arts in Industrial Psychology from Long Beach State College in 1962; right at the start of what we now call Industrial-Organizational Psychology. Dr. Bixler focused his studies on the design and analysis of American College Testing data to assess whether it was a useful tool to predict success in college. He recants how he spent countless hours tracking the academic progress of over 20 000 students by running punch cards through an IBM mainframe computer that filled an entire room; years before the age of the microprocessor and personalized computers. As we can only imagine nowadays, Dr. Bixler then managed to run discriminant function analysis of this summary American College Testing data by hand on an electronic calculator.

## The Apollo Space Program (1963–1966)

In May 1961, Pres. John F. Kennedy committed America to landing astronauts on the Moon by 1970 and christened it The Apollo program; described as one of the greatest technological achievements in human history. In 1963, Dr. Bixler’s career began as a Human Factors Specialist at NASA’s Apollo Space Program when Apollo 1 was in its initial stages of design with a focus on engineering divided into three dimensions: propulsion/navigation, electrical, and life support. Dr. Bixler recalls spending his days among approximately 400 desks lined up in an airport hangar in Los Angeles, California where he had been assigned to one of only 20 desks focused on “human factors,” as an industrial psychologist. The purpose of this task force was to identify human safety issues related to the Apollo mission. His division invited Neil Armstrong to test the capsule cabin, as he was shorter than the average US adult man; while engineers could not understand why this was important, they were tasked with building a plywood replica cabin and panel to strap Neil into the seat to calculate how fast he could reach the “escape button”; in fact, he could not even reach the control panel, given his height and being strapped into the seat. Dr. Bixler and the team played a literal life and death role in demonstrating to these engineers why paying attention to the “human factor” was important. The human factors group ended up with 3 divisions with Dr. Bixler’s focus on electrical safety. He was then relocated to the George C. Marshall Space Flight Center in Redstone Arsenal, Alabama, U.S. government’s civilian rocketry and spacecraft propulsion research center to manage the computer-driven mockup of the lunar roving vehicle and its related human factors. One of the tasks he was involved in was to monitor the astronauts in their pressurized suits while handling the lunar roving vehicle and whether they could move and maneuver properly on the moon’s surface. To further his training and better interface with engineers while continuing in the human factors area [1], Dr. Bixler pursued a Masters in Physiological Psychology at University of Arizona graduating in 1965. However, Dr. Bixler decided in 1966 to switch gears and leave behind the engineering world and pursue his doctorate.

## Something Called Physiological Psychology (1966–1970)

Dr. Bixler then transferred to the University of New Mexico in Albuquerque to pursue a PhD in Physiological Psychology, graduating in 1970. He joined the lab of Dr. Jack Rhodes, who was funded by the Holloman Airforce Base at NASA’s Mann Spacecraft Center. Dr. Rhodes had recently returned from a yearlong sabbatical in France studying sleep in animals with Dr. Michel Jouvet. It was this eventful coincidence that sparked Dr. Bixler’s interest in sleep. Dr. Bixler was sent to the Holloman Airforce Base as a lab technician to learn the effects of rapid decompression on chimpanzees and work with Dr. Martin Reite by monitoring the sleep of chimpanzees overnight and scoring the sleep records in the morning [2, 3], while Dr. Daniel F. Kripke was at the same base developing a machine to quantify sleep. Dr. Bixler recalls that during that time the first consensus standard for the staging of sleep in humans by Drs. Alan Rechstaffen and Anthony Kales were published in 1968, to realize that there was one variable missing from the type of sleep recordings they were doing in chimpanzees: the EMG, which allowed a proper scoring of paradoxical (rapid eye movement) sleep. Dr. Bixler became the only researcher in the lab interested in the application of animal studies on human sleep and he wanted to pursue such type of human studies. He established a lab in a hospital for individuals with intellectual disability that included clinical and sleep EEGs with a culmination of his work published in his dissertation focused on the visual evoked potentials and reaction time using tachistoscope in individuals with intellectual disabilities [4]. Given that the lab had its own digital equipment corporation computer, one of the first low-cost, mini-computers designed specifically for research labs, Dr. Bixler learned Fortran and then BASIC computer programming language and focused his interest on statistical analysis of sleep data. Later on, he personally met sleep research pioneer Dr. Jouvet, who inspired Dr. Bixler along with many generations of sleep researchers to explore the mysteries of sleep. He also studied alongside his contemporary Dr. John Orem, whose accomplishments would later include authoring the first edition of “Physiology in Sleep” together with Dr. Charles D. Barnes. After graduating, Dr. Bixler wanted to purse a postdoc. As he was the only one in the University of New Mexico lab doing work in humans and interested in sleep, Dr. Rhodes recommended Dr. Bixler to go to University of California, Los Angeles (UCLA) to work with Dr. Kales as a postdoc.

## UCLA’s Brain Research Institute (1970–1971)

Dr. Kales established the Sleep Research & Treatment Center (SRTC) in 1963 at UCLA’s Brain Research Institute. The SRTC was one of the first sleep labs in the country to provide both clinical and research overnight sleep studies. Dr. Bixler accepted a post-doctoral position in 1970 at Dr. Kales’ SRTC at UCLA along with his contemporary Dr. John Orem, who joined another lab. The focus of Dr. Bixler’s studies during his yearlong postdoc at UCLA’S SRTC were trials on hypnotic medications, which were primarily benzodiazepines that had not demonstrated efficacy as per the newer standards set-forth by FDA at the time. This was predictive of the future direction of the field of sleep pharmacology.

It is important to note here that during that year at UCLA, Dr. Kales and Dr. Bixler were the first researchers to include sleep items in a representative epidemiological study by adding five questions to the 1973 Los Angeles Metropolitan Area Survey (LAMAS). The importance of this decision will be presented below in great detail.

## Penn State College of Medicine (1971–2019)

**Figure 1.**
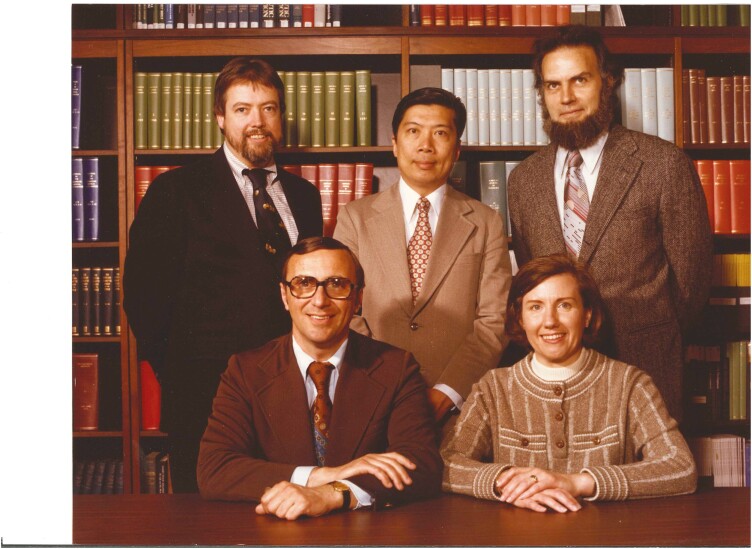
Core team that moved from UCLA to Penn State. Drs. Anthony Kales and Joyce Kales (front) moved the SRTC from UCLA to Penn State in 1971 and Dr. Edward O. Bixler (back right), a sleep researcher, and Dr. Ling Tan (back middle), a psychiatrist, moved with them. Also pictured in this photo is Dr. Fred Humphrey (back left), a psychiatrist, who joined the department in 1973.

**Figure 2.**
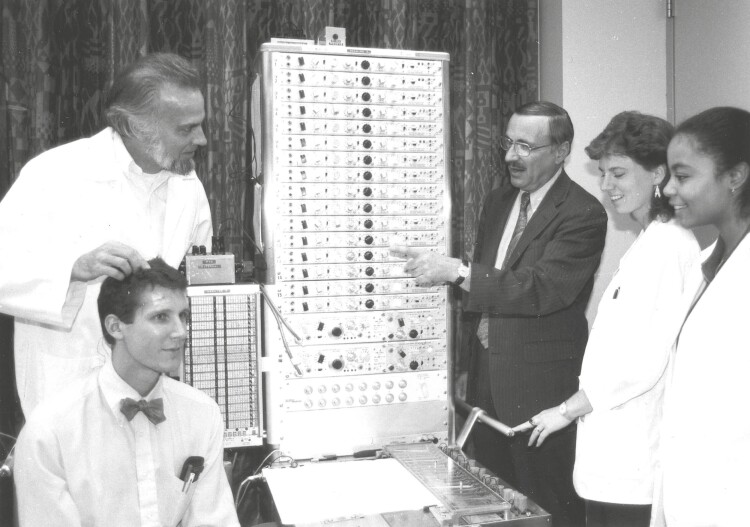
Teaching electroencephalography for sleep purposes at Penn State. Teaching and training in sleep and psychiatry was a focus of the SRTC and the Department, respectively, under Dr. Kales (second right) leadership. Dr. Bixler (standing left) participated in all data collection and training pertaining to the sleep lab. Here they are depicted with medical students.

**Figure 3.**
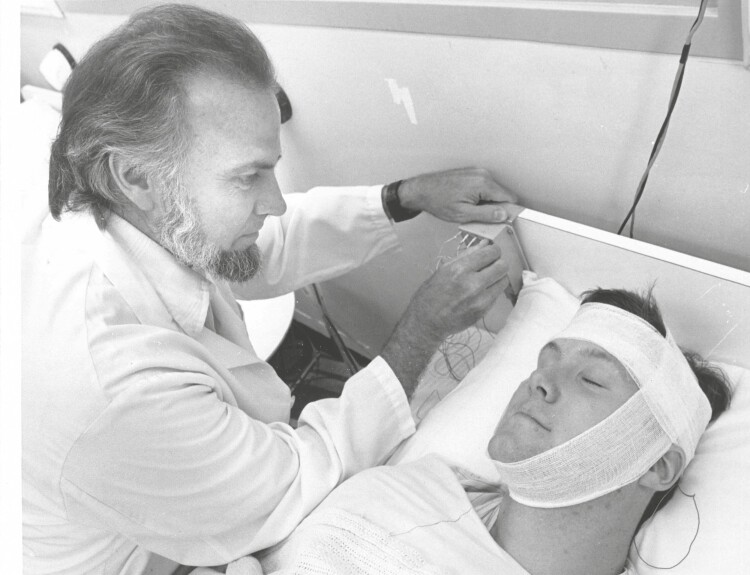
Managing the sleep lab at Penn State. Dr. Bixler was in charge of the sleep lab of the SRTC for decades, being responsible for all aspects from logistics, electrode soldering, acquisition, scoring, and interpretation of sleep data. He is pictured here with a patient who consented to have photos taken at the time. Photo by Bob Levy for Patriot News Co.

In 1971, Dr. Kales accepted the position of Chair of the Department of Psychiatry at the 1968-newly-founded Penn State University College of Medicine in Hershey, Pennsylvania. Dr. Kales did not simply move his career to Hershey, but the entire SRTC, its projects, vision, mission, and his closest clinicians and researchers (Figure 1). Dr. Bixler moved to Hershey and, as the Psychiatry Department needed to be built from the ground-up, he was pulled in many directions that involved most, if not all, research and administrative tasks (Figure 2). He played also a crucial role in establishing the Department’s missions. These included physically establishing the sleep lab, which consisted of six rooms and 12 beds, with a twofold goal of providing clinical care to patients while also collecting sleep data on research participants. Dr. Bixler, along with Ms. Mary Jeoco who scored all paper sleep records, was charged with summarizing all scored data; a herculean task given the “weighty” nature of these studies. In addition to running the technical aspects of the lab, Dr. Bixler was tasked with managing all sleep-related data and statistical analyses as well as supporting the Department’s mission for all faculty to be involved in research (Figure 3). Coming full circle from his early college days, these data were collected on punch cards that required statistical analysis only available at Penn State University Park main campus located 2 hours north of Hershey, requiring a couple day turnaround, except at the end of semester when the computer analysts were all too busy! Eventually, in the years to follow, the Department purchased digital equipment corporation desktops with SPSS and connected them with a coaxial cable to streamline statistical analysis. Dr. Bixler recalls “I was responsible for all research performed in the department when Dr. Kales was Chair; if the person had no experience, they spent a lot of time with me on Saturdays to see what they could do that was relevant to their work, as most were clinicians. I had to find out something that each faculty, medical student, or resident were interested in, so that they could develop some piece of research.” Readers are encouraged to refer to Dr.Kales’s Living Legends paper for a list of mentees for which Dr. Bixler was a contemporary or contributed to their career. Among them, Dr. Bixler recalls: “I spent a lot of time with Antonio (Dr. Vela-Bueno) and Soldatos (Dr. Constantin Soldatos), and later on with Alex (Dr. Vgontzas). I worked with Soldatos on how to record sleep, while he spent time with Kales writing papers. I worked with Antonio on translating our clinic questionnaire into Spanish and designing his epidemiological study in Madrid (Spain). Alex did not spend much time in the sleep lab but started working with the benzodiazepine data and clinical data on sleep apnea. Any data that was collected in the sleep lab, I was totally responsible for, all of it, all the steps from acquisition to interpretation. So, any trainee or faculty spent some time with me when they did research with sleep lab data. I recall also Drs. Nancy Collop and Dennis Charney, who were medical students at the time working with Dr. Kales. Also, I helped Dr. Dick Mattison, a child psychiatrist, and Dr. Susan Mayes, a child psychologist, get started with their research and data analysis.” It should come as no surprise then that Dr. Bixler eventually became the Department’s Vice Chair for Research. Dr. Bixler recalls “after Dr. Kales’s retirement, my role was similar but changed to support only those who asked for a research project, particularly residents. Then, when Dr. Alan Gelenberg became Chair and recruited Dr. Roger Meyer, we joined forces to focus on supporting and mentoring research for junior faculty in the department.” Dr. Bixler’s personality and unique character shone through in his training and mentoring of students and faculty. He is well known for his generosity towards others, his humble approach to science and his steadfast principle “stick to the data” no matter if the findings and their implications were mainstream or not. He was also well known for his Socratic-style, laconic, and humorous responses to young scientists’ questions such as “Dr. Bixler may I ask you a question?” and his response “you already did” or “I would like to discuss with you my research proposal” and his response “what is the question?” followed by “what data do you have?.” Dr. Bixler instilled in trainees and faculty a focus on the clarity of the scientific premise (question) and less on specific statistical methods.

Of note, a few years after establishing in Hershey, a group was created during a lunch meeting organized by the late Dr. Peter Hauri at the Edinburgh sleep conference in 1975 with discussions continuing later that year in Chicago. Dr. Bixler, who had been heavily involved in the Association for the Psychophysiological Study of Sleep (APSS), particularly during Drs. Kales and Vern Pegram’s tenure (1970–1972), was part of the leadership group that included Drs. William Dement, Thomas Roth, Ismet Karacan, Milton Kramer, David Kupfer, Howard Roffwarg, and Elliot Weitzman, who agreed that a new organization should be formed that would be sleep-center oriented with a strong clinical and research direction, which they named the Association of Sleep Disorders Centers, precursor of the American Sleep Disorders Association, later converted into the American Academy of Sleep Medicine [5]. While Dr. Bixler did not stay active in these latter clinical societies, he did in those that gave birth to the Sleep Research Society, of which he remains a member.

Led by Dr. Kales, Dr. Bixler’s research continued to focus on assessing the safety and efficacy of various new hypnotics compared to the barbiturates that were approved at the time as well as to comprehensively study several sleep disorders from a “whole patient” perspective. Dr. Bixler played a major methodological role in all of the clinical trials on short half-life and high affinity of benzodiazepines to explain their adverse effects (e.g. rebound insomnia, early morning awakening, anterograde amnesia) [6–22], and particularly those focused on their cognitive performance effects [23, 24], and also on the comprehensive clinical, polysomnographic, and psychometric study of chronic insomnia (i.e. its association with depression and personality profiles and not to PSG findings such as sleep apnea or nocturnal myoclonus), narcolepsy and other sleep disorders [25–33] conducted in Hershey until the late 1980s. Readers are encouraged to refer to Dr. Kales’s Living Legend paper in this series for a complete perspective on all this extensive, collaborative work.

**Figure 4.**
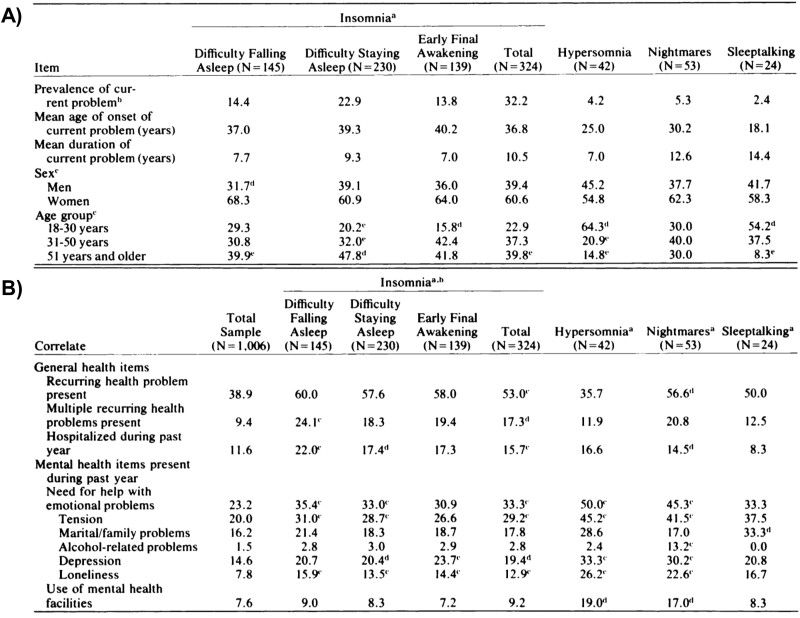
Key Findings of the Los Angeles Metropolitan Area Study. (A) This was one of the first epidemiological studies to systematically report on the prevalence of sleep disturbances, their age of onset, chronicity as well as sex and age differences. (B) The study also first reported on the prevalence of the same sleep disturbances as a function of the presence of chronic physical health problems, hospitalizations, and mental health problems, including the association of depression with insomnia symptoms in a non-clinical, general population sample. *Taken with permission from Bixler et al* [34].

Around 1977, after the LAMAS study data had been collected, processed, and cleaned, the sleep data and other health variables were analyzed and results were published by Dr. Bixler in 1979 [34]. As previously reviewed [35, 36], surveys of early cohort studies, such as the Framingham Heart Study, included issues like smoking, drinking, exercise, or diet but not sleep, which was not included until the 1980s. Indeed, sleep questions were also not included in the National Health Interview Survey, the National Health and Nutrition Examination Survey, the Cancer Prevention Study II, or the Nurses Health Study until 1977, 1982–1984, 1982, and 1986, respectively. Thus, Dr. Bixler’s paper on sleep disturbances and disorders in the LAMAS study is recognized as the first rigorous study in the field of “sleep epidemiology” [37], and became one of the most widely cited papers for that purpose during the 1980s and 1990s. The LAMAS study was conducted on a well-established representative sample of the LA metropolitan area (*N* = 1006) and was based on detailed census data, where investigators identified a house within a census area, and an interviewer was sent to knock on the door and go through the survey with the participants [34]. As shown in Figure 4, Dr. Bixler found an overall prevalence of 52.1% for a lifetime history (current or previous) of sleep disorders in adults. Specifically, he found a 42.5% prevalence of insomnia symptoms, 11.2% of nightmares, 7.1% of excessive sleep, 5.3% of sleep-talking, and 2.5% of sleepwalking. He also reported, with great detail at the time, how these sleep conditions were often chronic and usually started early in life. Notice how he seminally reported, in a nonclinical sample, an age of onset at 37 years old with a mean duration of 10 years for insomnia, while an age of onset at 25 years old with a mean duration of 7 years for hypersomnia; data that have passed the test of time, evidence and replication. Also, he reported that insomnia was more frequent in older people, particularly older women, and in people of lower educational socioeconomic status (Figure 4A); incipient data for the current revolution in sleep health disparities research. In addition, he confirmed in a general population sample, that insomnia, nightmares, and hypersomnia were more prevalent in individuals with frequent physical and mental health problems, particularly anxiety and depression (Figure 4B). Although Dr. Bixler admits that the study was statistically underpowered, there is no doubt that this was a seminal contribution to the sleep field.

Dr. Bixler’s newly sparked interest in epidemiology was also reflected in his early 1980s papers that used polysomnography (PSG) to describe the level of sleep apnea and myoclonus activity in samples of otherwise “good” sleepers recruited from the general population [38–43] as well as in patient populations not routinely studied at the time for their sleep with PSG, such as first noticing the increased prevalence of sleep apnea in patients with hypertension [44–46]. This early effort is pioneering as none of the national surveys mentioned above included objective sleep measures [35] and PSG was not included in epidemiological studies until the Wisconsin Sleep Cohort (WSC), the Sleep Heart Health Study (SHHS), and the Penn State Adult Cohort (PSAC) started recruiting study participants from 1989 to 1993, from 1995 to 1997 and from 1990 to 1999, respectively [35, 36].

### Sleep epidemiology: the PSAC (1990–1995; 1994–1999; and 1995–2004)

Dr. Bixler states that Dr. Kales purported three critical research principles: data accuracy, assessing the “whole individual” and “all aspects of sleep.” These principles were the basis to start conducting population-based studies that included PSG. Dr. Bixler decided to submit a grant proposal titled “Sleep Apnea: Age Effects on Prevalence & Natural History” to the National Heart, Lung and Blood Institute (NHLBI) in 1988 with the aim of identifying the prevalence of sleep-disordered breathing (SDB), including obstructive sleep apnea (OSA), as a function of age in men and assessing the natural history of the disorder in a subsample. This grant was scored but not funded. At the time, Dr. Marshall Jones of the Department of Behavioral Science and Dr. Bixler at the Department of Psychiatry were the only research statisticians at the Penn State College of Medicine. Shortly thereafter, Dr. Richard Landis was recruited as the first Director of Biostatistics and Epidemiology, now named Department of Public Health Sciences. Dr. Bixler asked Dr. Landis to review the grant and he incorporated several important changes in the sampling strategy; specifically, overweighting high-risk sleep apnea participants like National Health and Nutrition Examination Survey did for other conditions to assure that the targeted individuals were effectively recruited and to increase the precision of prevalence estimates. Dr. Landis also arranged for a meeting at the University of Michigan’s Epidemiology Department that provided crucial recruitment strategies and Dr. Bixler with a large binder of policies and procedures for epidemiological studies. It is there that Dr. Bixler had the good fortune to meet statistician Dr. Leslie Kish who had developed the Kish Grid, a method for selecting members within a household to be interviewed. After Dr. Kish’s review, this pre-call selection strategy was adopted and men from central Pennsylvania were recruited for an epidemiological study to determine the degree, if any, of SDB in the general population. The grant proposal was funded in the second round in January 1990.

In brief, the cohort study consisted of 4364 randomly sampled men selected from the local general population and stratified by age who were evaluated for risk factors for sleep apnea. Dr. Bixler recants “The world was different back then. Your phone number’s first three digits defined your general location, because all switches were mechanical and all connections were wired. We selected the physical area we wanted to sample based on those first three digits. We then randomly-select a fixed number of participants from the remaining four digits of the phone number. The task then was to call the given number. If the number was a business or was not operational we replaced it with another randomly selected number. If we were unable to get an answer, we did not replace the number. When the person answered, we then used the Kish strategy to identify the individual in the household to answer the interview. The interview was printed on a form that later could be scanned using the same method as the one developed for SATs. From the interview data we used the sampling strategy developed by Dr. Landis to identify the actual participant.” Of all men invited, 741 were evaluated in the sleep laboratory yielding a 67.8% response rate. Prior to recording participants in the sleep laboratory, a thorough history was obtained from each participant and a physical examination was completed. Also, a battery of more than 10 neurocognitive, personality, behavioral, and contextual tests were administered, which was at odds with sleep epidemiological studies of the time, and included Mini-Mental Status Examination, Symbol Digit Modality Test, Trail Making Test, Benton Visual Retention Test, Thurston Word Fluency Test, Minnesota Multiphasic Personality Inventory, Symptom Checklist-90, among many others. Those men entering into the first 4 years of the prevalence portion of the study who were found to have SDB were reevaluated yearly over a 5-year period in the natural history part of the study. The natural history portion also included 150 men previously studied in the sleep laboratory for SDB but who were not part of the random sample of 741 men in the prevalence study.

In 1993, following the successful recruitment rates with men, Dr. Bixler submitted a grant proposal with the same design and goals titled “Prevalence of Sleep Apnea in Women.” The proposal was scored and funded based on the first round of reviews in April 1994. In order to establish the prevalence in women with reasonable precision, the proposed prevalence determination employed a two-stage sample modified from the prevalence study in men in the following two ways: (1) an expanded telephone sample (*N* = 12 219 women) selected randomly from the local general population was evaluated for clinically relevant risk factors for SDB; and (2) a second sample (*N* = 1520) were selected randomly from the first sample based on higher risk for SDB, including menopausal status, and were invited to the sleep laboratory to determine the presence of any type of SDB. Of all women invited, 1000 were evaluated in the sleep laboratory yielding a 65.8% response rate. The same in-lab clinical procedures were employed prior to recording women in the sleep laboratory as in the men's study.

**Figure 5.**
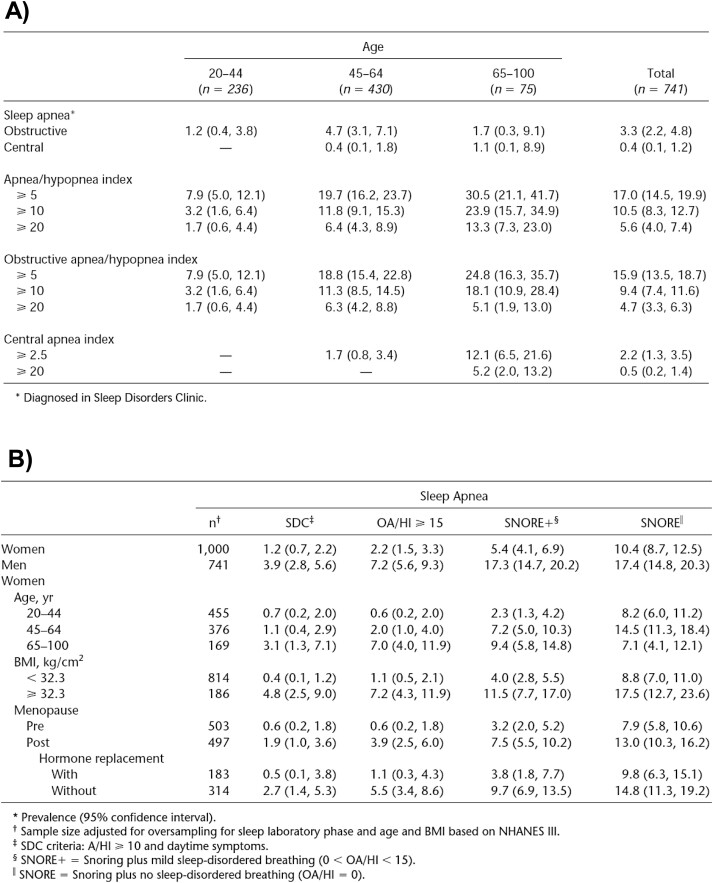
Prevalence of SDB in the Penn State Adult Cohort. (A) Prevalence of different types and levels of SDB as a function of age in men (*N* = 741). *Taken with permission from Bixler et al.* [48] (B) Prevalence of different types and levels of SDB in men (*N* = 741) and women (*N* = 1000) as well as a function of age, obesity, and menopausal and HRT status. *Taken with permission from Bixler et al* [49].

**Figure 6.**
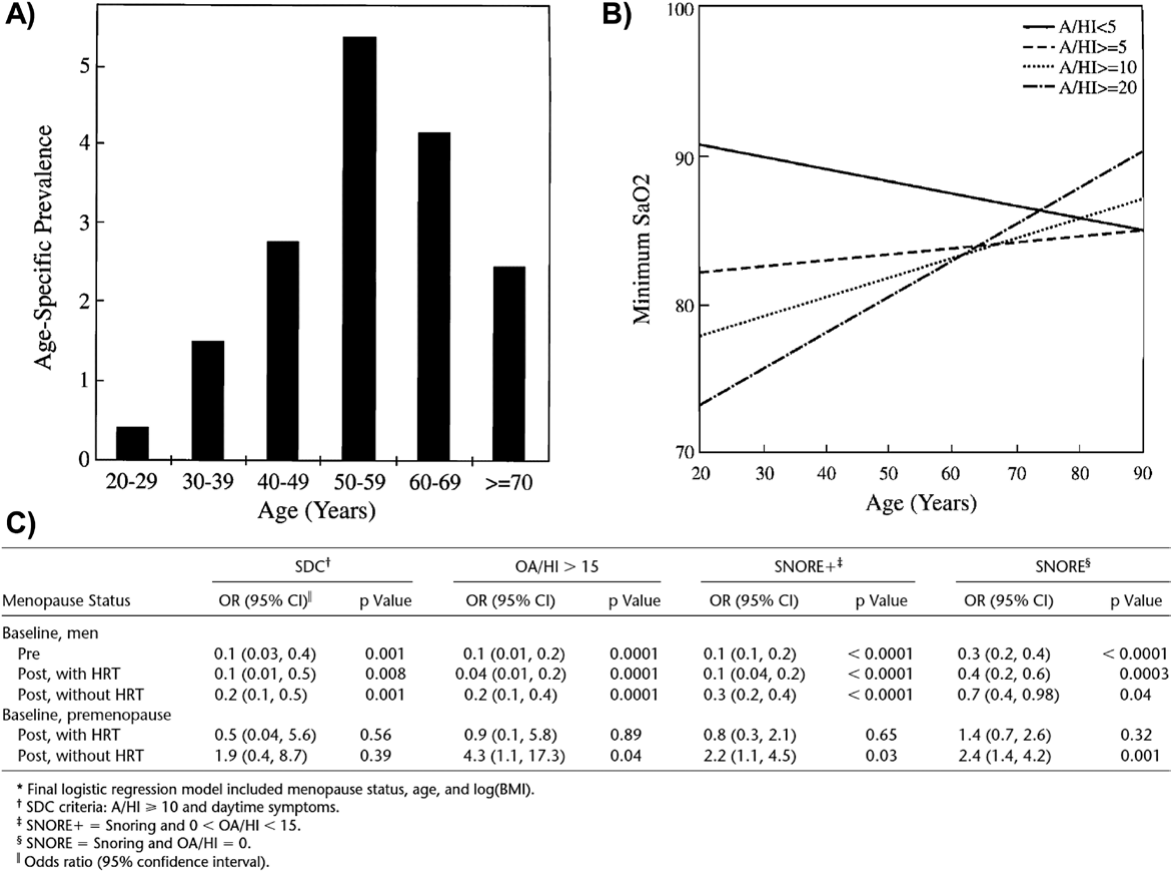
Effect of Age and Gender on SDB in the Penn State Adult Cohort. (A) The prevalence of OSA, defined as an OA/HI ≥ 10 plus the presence of daytime symptoms or comorbidities increases up to the fifth decade of life in men (*N* = 741). (B) The severity of OSA, as measured by the slope of minimum SaO_2_ and defined across four different levels of the AHI, was greater in younger men than older men (*N* = 741). *Taken with permission from Bixler et al*. [48] (C) Women, regardless of menopausal status, were significantly less likely to have any type of SDB compared to men (*N* = 1741); however, postmenopausal women not taking HRT were 4-times significantly more likely to have an OA/HI ≥ 15 compared to premenopausal women (*N* = 1000). *Taken with permission from Bixler et al.* [49].

Based on these two population-based samples of men and women, later named the PSAC, Dr. Bixler reported pioneering findings and replicating data. Of note, the analytic strategy was heavily influenced by Dr. Landis and his team of newly recruited statisticians, where Dr. TenHave was very helpful in improving the sampling weights [47] but statistically analyzed the data with a traditional strategy of slicing the data into separate cells. In contrast, when Dr. Hung Mo Lin was brought on board, she and Dr. Bixler took a slightly different approach to the PSAC data using continuous analysis [48, 49]. As shown in Figure 5, Dr. Bixler found that the prevalence of clinically defined OSA (i.e. AHI ≥ 10 and daytime symptoms or comorbidities such as excessive daytime sleepiness, hypertension, or other cardiovascular complication) was 3.3% in men (Figure 5A), while 1.2% in women (Figure 5B). Given the wide age range of the PSAC spanning from 20 to 88 years old, which contrasted with the narrower age ranges of the contemporary WSC (30 to 70 years old) and SHHS (40 years and older), Dr. Bixler was able to report on the important impact that age, and age-related factors (e.g. menopause), had on the prevalence and severity of SDB. As shown in Figure 6A, the highest prevalence of clinically defined OSA in men was in the middle-age group 45 to 64 years old, with a peak at the fifth decade of life [48]. In addition, the severity of SDB, as measured by the slope of minimum SaO_2_ and defined across four different levels of the AHI, was greater in younger men than older men (Figure 6B). As it pertained to women, they were indeed less likely to have any type of SDB, regardless of menopausal status, compared to men (Figure 5B). However, as shown in Figure 6C, the odds of SDB in women in any of the three categories of menopausal status were significantly lower compared to men, but less so in postmenopausal women not taking HRT. In fact, the odds of moderate-to-severe OSA in postmenopausal women not taking HRT were 4-fold compared to premenopausal women, even after adjusting for age and BMI [49]. The presence of OSA in premenopausal women and postmenopausal women taking HRT was associated exclusively with obesity [49]. These findings were later replicated in the WSC [50] and SHHS [51], although a potential “healthy user bias” effect for HRT on SDB was later studied in depth [52]. Nevertheless, as Dr. Bixler reported years later, women without sleep complaints do sleep physiologically better across all ages compared to men, reflected by lower stage 1 and higher slow wave sleep and sleep efficiency, while menopause in the absence of HRT in women is associated with prolonged sleep onset latency and decreased slow wave sleep above and beyond SDB [53].

**Figure 7.**
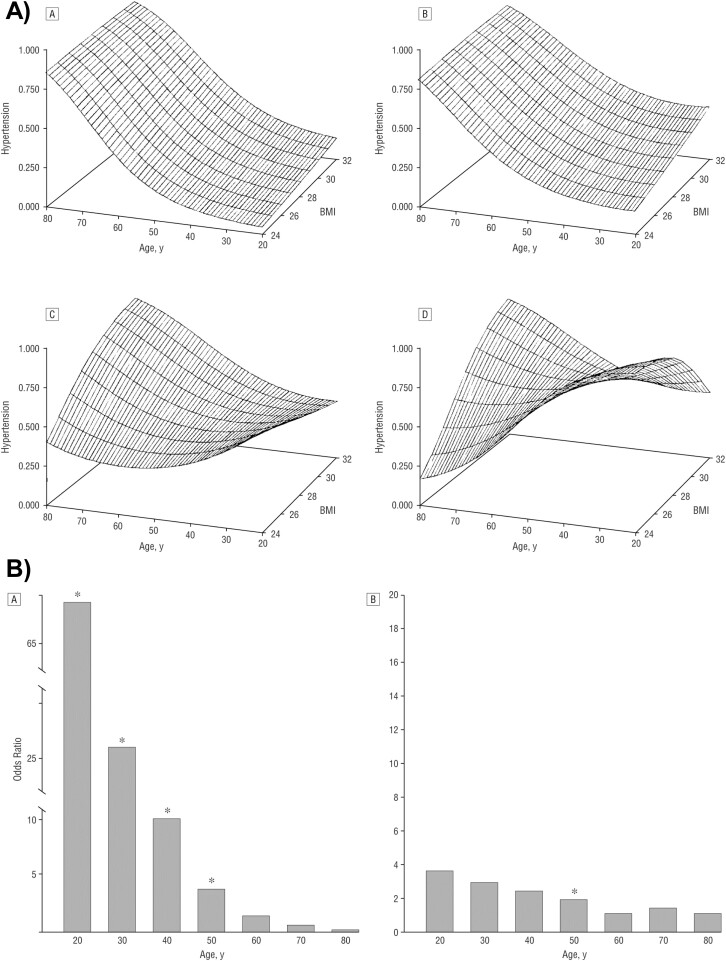
Association of SDB with Hypertension in the Penn State Adult Cohort. (A) Surface plots representing men without SDB [A], snoring [B], mild SDB [C], and moderate-to-severe SDB [D]. The shape of the distribution of the prevalence of hypertension in women alone (*N* = 1000) or in men and women combined (*N* = 1741) was almost identical to the distribution of the men alone shown in the surface plots above that depict the probability of having hypertension across age and BMI levels in men (*N* = 741). (B) The odds of hypertension associated with an OA/HI ≥ 15 (vs. no SDB) in men and women (*N* = 1741) were significantly increased in the second through fifth decade of life in those with overweight (BMI = 28) [A], while greatly diminished in those with obesity (BMI = 32) [B]; asterisks indicate *p*-value < 0.05. *Taken with permission from Bixler et al* [54].

In addition, Dr. Bixler also reported in the PSAC that SDB, even primary snoring, was independently associated with hypertension. The surface plots of Figure 7A depict the probability of having hypertension across age and BMI levels, and show that the association between SDB and hypertension was strongest in young and middle-aged adults, especially those of normal weight. Specifically, the odds of hypertension associated with moderate-to-severe OSA were greatly increased in the second through fifth decade of life in men and women who were overweight, while diminished in those who were obese, as shown in Figure 7B [54]. Taken together, the data presented in Figures 5–7 supported early on the hypothesis that SDB in older adults is less severe in terms of its degree of associated hypoxemia and impact on cardiovascular health, while SDB in young and middle-aged adults is more severe in terms of both health dimensions. These findings were the first to support and propose the position that sleep laboratory criteria (AHI) employed for the diagnosis of SDB and OSA should be age-adjusted; a position that has not yet been accepted by sleep scoring manuals or current nosology.

**Figure 8.**
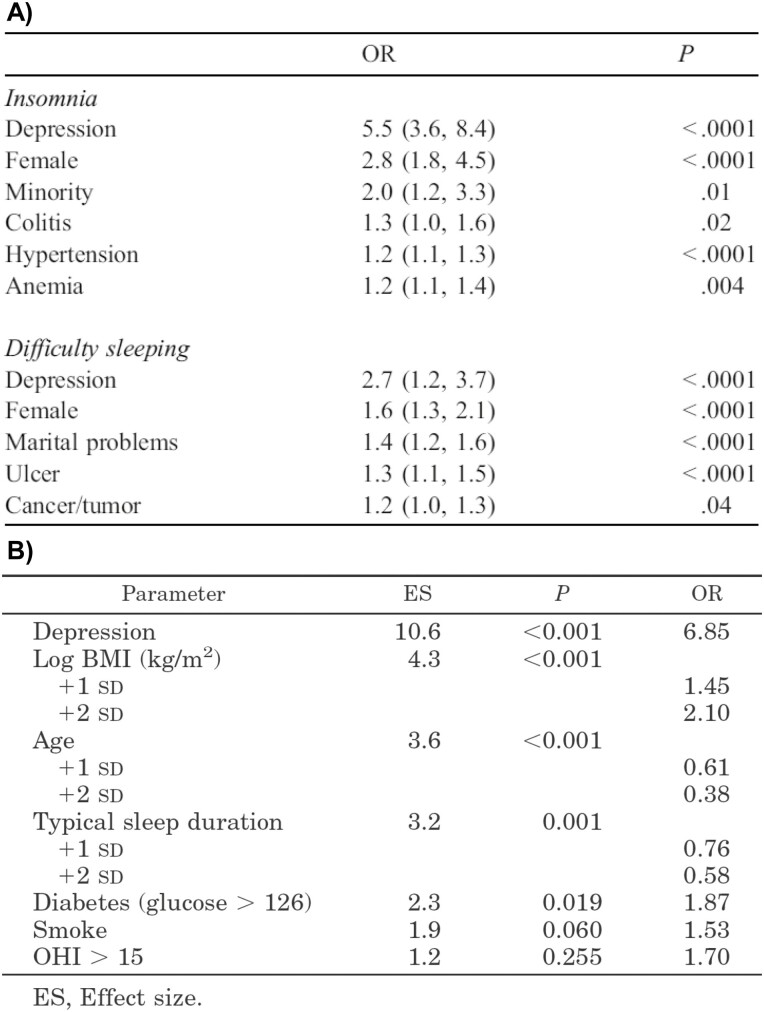
Prevalence of Insomnia and Hypersomnia in the Penn State Adult Cohort. (A) The prevalence of chronic insomnia, defined as a report of having insomnia lasting a year or longer, was 7.5% and was significantly higher in women and racial/ethnic minorities and in those with depression, colitis, hypertension or anemia, while the prevalence of sleep difficulty, defined as moderate-to-severe insomnia symptoms (i.e. difficulty initiating sleep, difficulty maintaining sleep, early morning awakening, and/or nonrestorative sleep) was 29.9% and was significantly higher in women and in those with depression, marital problems, ulcer or cancer/tumor. *Taken with permission from Bixler et al*. [55] (B) The prevalence of EDS, defined as moderate-to-severe complaints of feeling drowsy or sleepy most of the day but managing to stay awake and/or having any irresistible sleep attacks during the day, was 8.7% and was significantly higher in younger adults and in those with depression, obesity, diabetes or those reporting shorter sleep duration or being a smoker. *Taken with permission from Bixler et al.* [56]*.*

As Dr. Bixler collected data in the PSAC on multiple other sleep and health dimensions beyond SDB, he was also able to report on the prevalence of other sleep disorders and chief complaints. As shown in Figure 8A, Dr. Bixler reported that the prevalence of “chronic insomnia,” defined as a report of having insomnia with a duration of one year or longer (i.e. insomnia disorder), was 7.5% and was significantly higher in women and racial/ethnic minorities and in those with depression, colitis, hypertension or anemia, while the prevalence of “difficulty sleeping,” defined as moderate-to-severe difficulty initiating sleep, difficulty maintaining sleep, early morning awakening and/or nonrestorative sleep (i.e. insomnia symptoms), was 29.9% and was significantly higher in women and in those with depression, marital problems, ulcer or cancer/tumor [55]. This approach to defining insomnia both as a condition and by its symptoms deviated from his prior LAMAS study and was contemporary to the work of his colleague Dr. Maurice Ohayon [57], later followed by others building larger population-based insomnia cohorts such as that of Dr. Charles Morin [58].In addition, Dr. Bixler reported on the prevalence and risk factors of EDS, defined as moderate-to-severe complaints of feeling drowsy or sleepy most of the day but managing to stay awake and/or having any irresistible sleep attacks during the day. The prevalence of EDS was 8.7% and, as shown in Figure 8B, it was significantly higher in younger and older adults (U-shape) and in those with depression, obesity, diabetes or those reporting shorter sleep duration or smoking [56]. About the same time as these publications, Dr. Bixler’s team, and in particular Dr. Susan L. Calhoun, was charged with conducting follow-up telephone surveys of the PSAC, collecting data on 1395 of the original men and women 5 to 10 years later, yielding a response rate of 80.1%. These data were the source of many longitudinal studies using the PSAC over the years by the team of researchers at the SRTC. Indeed, although we have highlighted Dr. Bixler’s most important scientific contributions to the field with the PSAC, as he established this cohort study and assessed systematically for multiple sleep and general and mental health domains, he paved the way for others to foster their careers and research ideas, particularly Dr. Bixler’s long-term colleague, Dr. Alexandros N. Vgontzas. Dr. Bixler recalls that “Alex joined the Department as a psychiatry resident in 1985 and was rapidly evolving within the Department at the time that I was recruiting participants for the adult cohort. From 1991 until 2007, he was in charge of the Sleep Clinic of the SRTC, where he assessed all sleep disorders, including many patients with OSA. Looking back on Alex’s impact, we had a strong synergistic relationship. As he developed his model of OSA as a manifestation of the metabolic syndrome (i.e. visceral obesity, inflammation, and insulin resistance) [59, 60], we were able to confirm it with epidemiological data. Alex was critical in the interpretation and clinical relevance of the findings of the PSAC, such as the age effect on OSA’s symptomatology, that AHI alone cannot explain the association of OSA with EDS, and the importance of the protective role of female hormones on the development of OSA in women. At some point he began using the adult cohort data directly, particularly to test his insomnia model [61–63].” Those multiple other studies [64–81] will be the source, we are sure, of future sleep biographies.

### Sleep epidemiology: The Penn State Child Cohort (2000–2005; 2010–2013)

Inspired by life-course epidemiology, Dr. Bixler brought-in Drs. Susan L. Calhoun and Susan D. Mayes, both child psychologists, to inform the type of developmental, clinical, and psychometric measures needed to oversample for risk factors, such as ADHD, in order to establish the prevalence and clinical significance of pediatric SDB with reasonable precision. Dr. Bixler submitted a small grant proposal titled “Sleep in Children with Attention Deficit Disorder” to collect preliminary data in December 1998. He proposed a protocol similar to that used to establish the prevalence and clinical significance of SDB in the PSAC and the grant proposal titled “Prevalence of SDB in Children*”* was funded in August 2001. The study, later named the Penn State Child Cohort (PSCC), employed also a two-phase protocol; first, a questionnaire completed by the parents of every child enrolled in local elementary schools assessed general sleep, behavior, and learning problems with 5740 returned (80% response rate), a process in which Dr. Calhoun was also critical in making it a success; second, children (*N* = 1000) were selected randomly from the first sample based on stratification for grade, sex and risk of SDB from the parent’s returned questionnaires. In total, 700 children were evaluated in the sleep laboratory, yielding a 70% response rate. The 700 children underwent a thorough clinical history and physical examination, including a pediatric ENT and pulmonary evaluation, review of school records, cognition, and behavior were assessed using standardized measures and urine and saliva samples were collected in addition to an overnight PSG.

**Figure 9.**
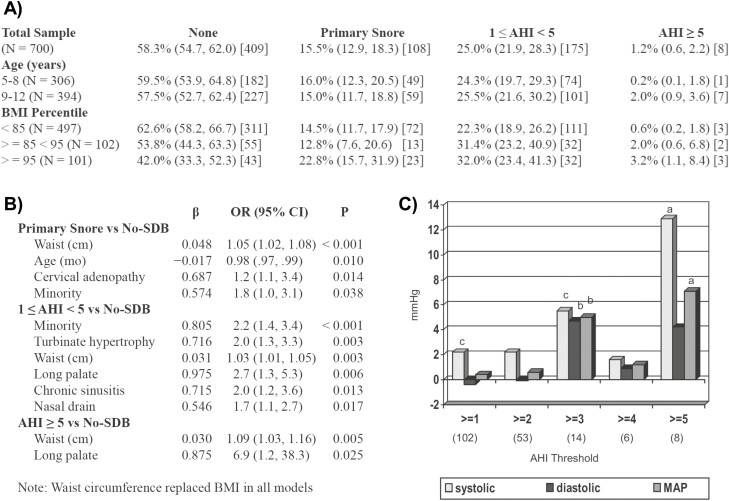
Prevalence of SDB, Risk Factors and Association with Hypertension in the Penn State Child Cohort. (A) The prevalence of primary snoring was 15.5%, that of mild SDB was 25.0%, and that of moderate-to-severe SDB was 1.2% in school-aged children (*N* = 700), and was significantly higher in those with overweight (85 ≤ BMI < 95) and/or with obesity (BMI ≥ 95); data are prevalence, 95% confidence interval (CI), and (sample size). (B) While different demographic, constitutional, and upper-airway anatomic risk factors emerged for different types and levels of SDB in children, central obesity, as measured by waist circumference, was consistently associated with SDB at all levels of severity; data are regression coefficient (β) odds ratio (OR), 95% CI, and *p*-value (P). *Taken with permission from Bixler et al*. [82] (C) Systolic blood pressure, diastolic blood pressure and mean arterial pressure (MAP)levels adjusted for age, sex, and height percentile in 700 school-aged children with different levels of SDB (i.e. AHI thresholds ranging from 1 through 5) compared to children without SDB (i.e. AHI < 1); a = *p*-value < 0.001, b = *p*-value < 0.05, c = *p*-value < 0.10); sample size for each subgroup presented in parenthesis. *Taken with permission from Bixler et al* [83].

As shown in Figure 9A, Dr. Bixler established the prevalence of 1 ≤ AHI < 5 to be 25.0% and that of AHI ≥ 5 to be 1.2% in the PSCC. As shown in Figure 9B, risk factors for SDB in these school-aged children included a higher waist circumference, having nasal abnormalities (e.g. chronic sinusitis/rhinitis), and identifying as a racial/ethnic minority per parent report, particularly Black/African American. The strong linear relationship between waist circumference and BMI across all degrees of severity of SDB suggested that, similar to adults, metabolic factors were among the most important risk factors for SDB in children [82]. As shown in Figure 9C, Dr. Bixler was also able to report a significantly elevated systolic blood pressure associated with an AHI ≥ 1 (2.9 mm Hg), AHI ≥ 3 (7.1 mm Hg), and AHI ≥ 5 (12.9 mm Hg), an association that remained significant even after adjusting for age, sex, race/ethnicity, BMI percentile or waist circumference, sleep efficiency, percentage of rapid eye movement sleep, and snoring. This was one of the first epidemiological studies to support that SDB is significantly associated with higher levels of systolic blood pressure in children aged 5 to 12 years even after adjusting for various important confounding factors [83].

**Figure 10.**
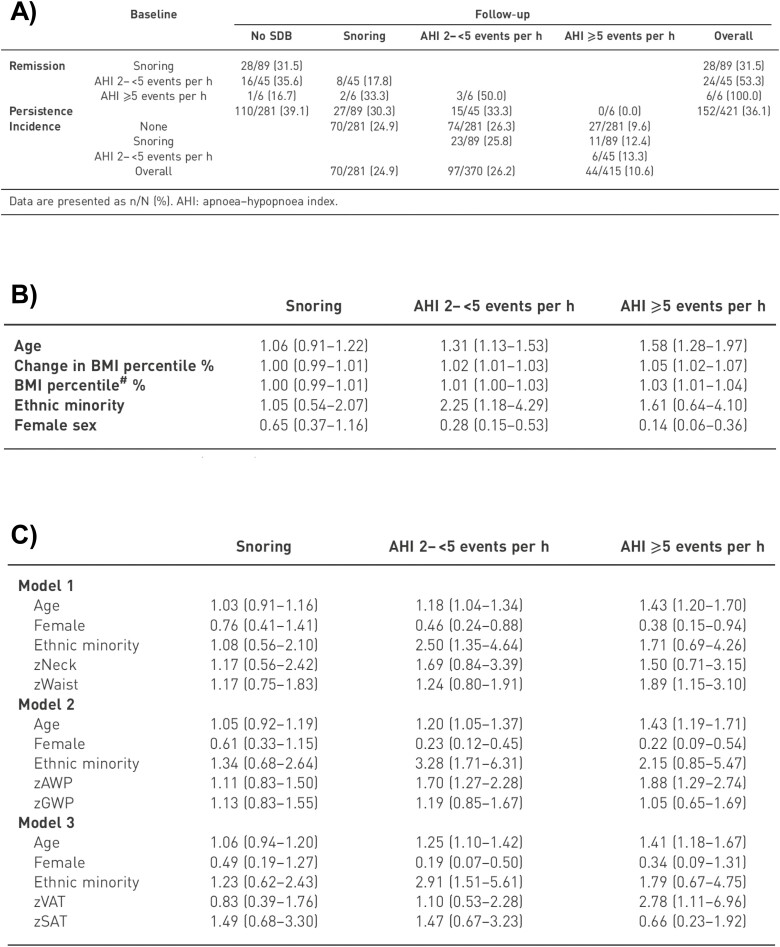
Natural history of SDB in the Penn State Child Cohort. (A) Persistence, remission, and incidence rates of SDB in school-aged children transitioning to adolescence (*N* = 421); data are n/N (%). (B) Risk factors independently associated with different levels of SDB in adolescence; data are odds ratio (OR), and 95% confidence intervals (CI). C) Systematic assessment of detailed body fat distribution and composition variables with different levels of SDB in adolescence; data are OR (95% CI); z, z-transformed; AWP, android/whole-body fat-mass proportion; GWP, gynoid/whole-body fat-mass proportion; VAT, visceral adipose tissue; SAT, subcutaneous adipose tissue. *Taken with permission from Bixler et al.* [84].

Dr. Bixler submitted a follow-up grant proposal titled “Sleep and Cardio-Metabolic Risk Factors: A Prospective Study of Adolescents” with Dr. Duanping Liao from Public Health Sciences as his co-PI that started in May 2010. Of the 700 children, 421 (60% response rate) returned 6 to 13 years later from 2010 to 2013 (median 7.4 years of follow-up) for a reexamination in the sleep lab for a repeat of their baseline study and the addition of in-lab dual x-ray absorptiometry (DEXA) scan and at-home actigraphy. A single fasting blood draw, for the assessment of plasma interleukin-6 (IL-6), IL-6 soluble receptor (IL-6 sR), tumor necrosis factor alpha (TNFα), tumor necrosis factor receptor 1A (TNFR1), C-reactive protein (CRP), leptin, and adiponectin levels, was added based on Dr. Vgontzas’ work on inflammation and role as long-term collaborator. Also, DNA for gene-wide analysis, 39-hour Holter EKG, and air pollution monitoring were added based on the incipient personalized medicine institute at Penn State and Dr. Liao’s expertise in environmental cardiovascular epidemiology. The PSCC became a longitudinal cohort study covering two developmentally distinct periods, allowing for examining the natural history of several sleep disorders, with a primary focus on SBD. As shown in Figure 10A, Dr. Bixler reported the remission of childhood OSA, defined as an AHI ≥ 2 events per hour of sleep, to be 52.9% in the transition to adolescence [84]. No children with moderate-to-severe OSA (AHI ≥ 5) persisted with the condition, while 50.0% partially remitted to mild OSA (2 ≤ AHI < 5) and the other half fully remitted (AHI < 2) in the transition to adolescence [84]. The incidence of mild OSA (AHI ≥ 2) in adolescence in those without childhood OSA (AHI < 2) was 36.5%, while the incidence of moderate-to-severe OSA (AHI ≥ 5) in adolescence in those without childhood SDB was 9.6%, in those with childhood snoring 12.4% and in those with mild childhood OSA was 13.3%. As shown in Figure 10B, the key risk factors for adolescent SDB, particularly incident moderate-to-severe OSA, were similar to those found in adults; namely, male sex, older age, and obesity [84]. Moreover, the study reported novel cross-sectional findings that the amount of DEXA-measured visceral adipose tissue was strongly associated with SDB in adolescence (Figure 10C), a finding consistent with studies in adults. In summary, Dr. Bixler’s longitudinal study confirmed that prepubertal OSA tends to resolve naturally during the transition to adolescence in a large proportion of children, and that pediatric snoring and mild SDB do not appear to be strongly associated with progression to more severe SDB during this developmental period. In addition, the study confirmed that adolescence is a critical developmental period for the onset of moderate-to-severe OSA, driven by similar risk factors as those identified in adults, particularly visceral obesity [84].

In November 2007, Dr. Bixler hosted a symposium on sleep epidemiology in youth and adults, including speakers such as Drs. Stuart Quan, Susan D. Mayes, Susan L. Calhoun, Duanping Liao, Terry Young, Naresh M. Punjabi, Donald L. Bliwise, Alexandros N. Vgontzas, Antonio Vela-Bueno, and Maurice Ohayon, that covered all topics from childhood SDB to adult insomnia in cohort studies [85]. Similarly to the PSAC, Dr. Bixler’s effort and persistence in establishing the PSCC allowed for multiple other investigators to further their career development and make their own contributions to the sleep field as reflected in the many publications that have aroused from it over the years [86–125], some of which were critical to serve as preliminary data for future funding efforts.

### Sleep epidemiology: passing the baton

One of the most significant contributions to science that a researcher and scholar embodies as they approach retirement is paving the way forward for others to continue with their scientific legacy. Dr. Bixler accomplished this by not only leaving behind a physical legacy at Hershey, but by having the foresight to plan and execute the “passing of the baton.” In 2013, Dr. Bixler supported Dr. Julio Fernandez-Mendoza in his desire to systematically track the cause of death of the PSAC by retrieving data from the National Death Index of the Center for Disease Control. The proposal titled “Interplay of Short Sleep Duration and Cognitive Impairment on Cardiovascular Disease and Stroke Mortality” was funded by the American Heart Association in April 2014, resulting in several primary publications and other secondary studies [126–131]. Dr. Bixler also supported Dr. Fernandez-Mendoza in his goal to follow-up the PSCC as young adults and add other health measures and dimensions. The grant proposal titled “The Penn State Child Sleep Cohort: Cardiometabolic and Neurocognitive Risk in Young Adulthood” was funded by the National Heart Blood and Lung Institute in August 2018, successfully surveying 520 of the original 5–12-years-old children as young adults 18-to-32-years-old and actively recruiting them into the sleep lab at ages 20 or older (about 280 as we type), providing data on the developmental trajectories of insomnia symptoms from childhood through adolescence into young adulthood [132, 133]. In addition, Dr. Bixler supported Dr. Fernandez-Mendoza’s effort to deeply analyze the PSG records of all participants in the PSCC as children (*N* = 700) and adolescents (*N* = 421) and extract sleep EEG biomarkers. The grant proposal titled “Sleep Cortical Dynamics and Neurobehavioral Risk in Children and Adolescents: A Longitudinal Study” was funded by the National Institute of Mental Health in April 2019, resulting in several publications on slow wave activity, sleep spindles and odds ratio product from age 5 to 23, cross-sectionally and longitudinally, and on their association with neurodevelopmental and emotional disorders [134–137]. Dr. Bixler reflects that “when Julio (Dr. Fernandez-Mendoza) took over, he expanded the depth of the cardiovascular outcomes with ultrasounds, brought the cognitive and behavioral assessments forward, and added new dimensions by extracting the sleep EEG biomarkers and getting samples of the gut. Julio also added another new dimension, where he is bringing-in more students than ever needing a thesis or dissertation and addressing a unique question from the two cohorts relevant to their background. He has expanded the data into teaching and training.”

## Retirement and Beyond (2019–present)

From all his contributions above, including the legacy left at the SRTC when he retired in 2019, Dr. Bixler recants “The design of all studies was influenced by many at Penn State. The basic design of these studies was influenced by Dr. Kales, i.e. ‘study the whole patient and all dimensions of sleep’. Thus, we collected data covering sleep disorders in general, with a focus on SDB. The 2-Sues (Drs. Calhoun and Mayes) developed the assessment of the cognitive and behavioral dimensions and were critical in recruiting participants, Alex [Dr. Vgontzas] added the assessment of metabolic markers and the theoretical models of OSA and insomnia, Duanping (Dr. Liao) added the cardiovascular and air quality dimensions to the adolescence, and Julio (Dr. Fernandez-Mendoza) added the deeper phenotyping with sleep EEG biomarkers to the children and adolescents and with cardiac ultrasounds and other measures to the current young adult follow-up. In summary, what I am most pleased about is that I believe that we have been able to add a better overall understanding of several sleep disorders and each person that contributed to the design was able to use data from these cohorts to further developing their own theoretical models.” We all agree Dr. Bixler, that you are a Living Legend, and along with the entire sleep research community are grateful to you for your pioneering vision and life-long dedication to our field.

## Biography

Edward O. Bixler, PhD, is Emeritus Professor of Psychiatry and Behavioral Health (retired) at the Pennsylvania State University College of Medicine (PSCOM). During his 40-year career, he co-directed the Sleep Research & Treatment Center at PSCOM, taking care of all of its technical logistics, infrastructure, technology, and staffing, until the two clinical sleep labs in Hershey were merged into a single venture in 2008. Thereafter, he was responsible for funding and securing two bedrooms in PSCOM’s Clinical Research Center to perform sleep studies for research purposes only. While at PSCOM, he also participated in the creation of the American Sleep Disorders Association and in systematically and comprehensively studying hypnotic medications and sleep disorders. He also published the first rigorous epidemiological study on sleep disorders. Subsequently, he established two cohort studies that included sleep lab recordings, which were later named the PSAC and the PSCC. The continuing success of these cohort studies was brought up by the role of his key co-investigators, Drs. Alexandros N. Vgontzas, Susan L. Calhoun, Duanping Liao, and Julio Fernandez-Mendoza, as well as long-time project manager, Ms. Carrie Criley. Throughout these years he helped maintain research programs at both basic science and clinical levels for both the sleep division and the Psychiatry department as a whole in his role as Vice-Chair for Research until his retirement in 2019. Dr. Bixler not only contributed significantly to sleep science but left a legacy behind that remains “alive-and-kicking” in Hershey. He gives name to one of the Faculty Endowments in the Department of Psychiatry and Behavioral Health created by Dr. Anthony Kales, the *“Edward O. Bixler, Ph.D., Professorship in Psychiatry,”* which is currently held by Dr. Fernandez-Mendoza.

## Data Availability

No new data were generated in support of this paper, which is essentially a review article.

## Notes

*This paper is part of the Living Legends in Sleep Research series, which is sponsored by Idorsia Pharmaceuticals and Jazz Pharmaceuticals.*
